# Retrograde Intrarenal Surgery in Patients Who Previously Underwent Open Renal Stone Surgery

**DOI:** 10.1155/2015/198765

**Published:** 2015-08-18

**Authors:** Erdal Alkan, Ali Saribacak, Ahmet Oguz Ozkanli, Mehmet Murad Başar, Oguz Acar, Mevlana Derya Balbay

**Affiliations:** ^1^Department of Urology, Memorial Sisli Hospital, Okmeydani, Sisli, 34385 Istanbul, Turkey; ^2^Department of Urology, Izmit Konak Hospital, Kocaeli, 41100 Izmit, Turkey; ^3^Department of Anesthesiology, Memorial Sisli Hospital, Okmeydani, Sisli, 34385 Istanbul, Turkey

## Abstract

*Purpose*. To ascertain whether retrograde intrarenal surgery (RIRS) is as effective in patients treated previously with open renal stone surgery (ORSS) on the same kidney as in patients with no previous ORSS. *Methods*. There were 32 patients with renal stones who had previous ORSS and were treated with RIRS in the study group (Group 1). A total of 38 patients with renal stones who had no previous ORSS and were treated with RIRS were selected as the control group (Group 2). Recorded data regarding preoperative characteristics of the patients, stone properties, surgical parameters, outcomes, SFRs (no fragments or small fragments <4 mm), and complications between groups were compared. *Results*. Mean age, mean BMI, mean hospital stay, and mean operative time were not statistically different between groups. Mean stone size (10.1 ± 5.6 versus 10.3 ± 4.2; *p* = 0.551) and mean stone burden (25.4 ± 14.7 versus 23.5 ± 9.9; *p* = 0.504) were also similar between groups. After the second procedures, SFRs were 100% and 95% in groups 1 and 2, respectively (*p* = 0.496). No major perioperative complications were seen. *Conclusion*. RIRS can be safely and effectively performed with acceptable complication rates in patients treated previously with ORSS as in patients with no previous ORSS.

## 1. Introduction

The surgical techniques in the treatment of stone disease have dramatically changed in the last 20 years [1]. There has been a significant decrease in the number of patients requiring open renal stone surgery (ORSS) due to the technological developments in the field of urologic surgery. Nowadays shock wave lithotripsy (SWL) and percutaneous nephrolithotomy (PNL) are recommended as the first line treatment modalities in the management of renal stones <20 mm and >20 mm, respectively [2]. However, ORSS has been performed in 0.47–2% in select patients [1].

Since stone recurrence rate within 5 years is about 50%, multiple interventions may be needed for patients with stone disease [3]. Reoperation in patients with previous open renal stone surgery (ORSS) would be difficult due to retroperitoneal scarring around the kidney and distortion of the pelvicaliceal anatomy. Eventually, reoperation may be associated with a longer operative time, higher complication, and lower success rates. There are many studies about PNL in patients who previously underwent ORSS [4–7]. But there is only one study on retrograde intrarenal surgery (RIRS) in patients who previously underwent ORSS in the English literature [8]. In order to ascertain whether RIRS is as effective in patients treated previously with ORSS on the same kidney as in patients with no previous ORSS, we retrospectively compared our groups including stone properties, operative parameters, and postoperative results.

## 2. Materials and Methods

This study was designed as a retrospective controlled study and included patients who underwent RIRS between December 2007 and January 2015. There were 32 patients with renal stones who had previous ORSS and treated with RIRS in the study group (Group 1). A total of 38 patients with renal stones who had no previous ORSS and treated with RIRS were selected as the control group (Group 2). First 4-5 patients from each year (2008–2015) who were treated with RIRS but did not undergo ORSS were picked out and put together to form the control group. Patients with renal stones together with ureteral stones were also included in this study. All patients were preoperatively evaluated by CT scan with stone protocol to define the total stone burden and collecting system anatomy. Stone burden was calculated by measuring the maximum stone dimension in cases with single stone or sum of dimensions in cases of multiple stones. Recorded data regarding preoperative characteristics of the patients (gender, age), stone properties (stone number, size, and burden), surgical parameters (operative time, use of stents, and basket), outcomes, Stone Free Rates (SFRs), and perioperative complications were collected. Prior to the procedure, informed consent was obtained from all patients.

All procedures were done in a standard lithotomy position under general anesthesia. In patients with intrarenal stones and concomitant middle or lower ureteral stones, a semirigid ureteroscope (8/9.8F Olympus, Tokyo, Japan) was used first for the treatment of ureteral stones. RIRS was performed by three experienced surgeons (each surgeon performed at least 100 RIRS procedures) using URF P-5 flexible ureteroscope (Olympus, Tokyo, Japan) or Cobra flexible dual-channel ureteroscope (Richard Wolf, Knittlingen, Germany) according to its availability. A ureteral access sheath (Flexor ureteral access sheath 12/14F 35 cm; Cook Medical, Bloomington, IN, USA) was used routinely in order to access to the collecting system easily and decrease the intrarenal pressure. In cases where the ureteral access sheath or flexible ureteroscope without access sheath could not be advanced due to ureteral pathologies such as ureteral stricture, a ureteral stent was inserted into the ureter and the procedure delayed for 2 or 3 weeks. Holmium YAG laser (Sphinx, Lisa Laser, 30 watts, Katlenburg, Germany) in combination with a 200 *μ*m (Litho Fib, Lisa Laser, Katlenburg, Germany) or 272 *μ*m (Flexi Fib, Lisa Laser, Katlenburg, Germany) laser fibers was set at an energy level of 0.6–2.2 J and at a rate of 5–12 Hz. This is the only available laser source at our hospital. Holmium laser was used for stone fragmentation in all cases. All stones were fragmented until roughly all get smaller ⩽4 mm in size, and relatively larger fragments were removed with a nitinol basket (Ngage nitinol stone extractor 2,2F 115 cm basket; Cook Medical, Bloomington, IN, USA) according to surgeon's discretion. Endoscopically, intraoperative success was defined as extraction of all stone fragments or laser lithotripsy of all stones to less than 4 mm fragments. At the end of the operation, an internal stent (usually 4.8F 26 cm) was inserted according to surgeon's discretion, which was removed under local anesthesia using a flexible ureteroscope in about 1 month of surgery. Residual fragments were assessed with noncontrast CT two months after internal stent removal. Success rate was defined as no fragments or the presence of clinically insignificant residual fragments (CIRF) smaller than 4 mm in the urinary system [2, 9]. All postoperative complications were recorded according to the Clavien-Dindo classification system [10].

### 2.1. Statistical Analysis

All analyses were performed using SPSS version 16.0 (Statistical Package for Social Sciences for windows; Chicago, IL, USA). The measurement data were expressed as mean ± standard derivation. Age, BMI, stone number, stone size, stone burden, operative times, and hospitalization times were compared by using Mann-Whitney *U* test. Additionally, use of ureteral access sheath and basket catheter, internal stent placement, SFR, and complication rates were compared by using Pearson Chi- Square test. *p* value of <0.05 was considered statistically significant.

## 3. Results

A total of 41 RIRS procedures performed on 32 patients were included in group 1. While 27 of 32 (85%) patients required a single procedure, 3 (9%) patients were treated with 2 procedures. Two (6%) more patients with bilateral renal stones required a total of 8 procedures (two procedures for each kidney) in group 1. On the other hand, RIRS was performed in 34 of 38 (90%) patients as a single procedure in group 2. Additionally second-session RIRS was performed in 2 (5%) patients, and bilateral RIRS was done in another 2 (5%) patients. As a result, a total of 41 and 42 RIRS procedures performed on 32 and 38 patients were included in groups 1 and 2, respectively. RIRS were performed due to back pain (54%), renal colic (23%), recurrent urinary tract infections (11%), persistent hematuria (9%), and patient preference (3%). Lower ureteral stones together with renal stones were present in 3 and 5 patients in groups 1 and 2, respectively. These stones were treated at the same session. Patient demographics and preoperative stone characteristics are shown in Table 1.

Intraoperative and postoperative comparisons between groups are summarized in Table 2. A ureteral access sheath could be placed in all patients but 2 in group 1. RIRS was successfully completed without access sheath in these cases. However, although a ureteral access sheath could be placed in another 2 patients in group 1, renal stones could not be reached due to concomitant ureteropelvic stenosis. Endopyelotomy and internal stent placement were performed in these patients and RIRS was successfully done after 30 days. A ureteral access sheath could not be placed in 5 (12%) patients in group 2. While 3 patients were treated without access sheath, 2 patients were stented and RIRS was delayed for 15 days. After all procedures, SFRs were 100% and 95% in groups 1 and 2, respectively (*χ* ^2^ = 1.747; *p* = 0.496). Although 24 (71%) and 30 (75%) renal units were truly stone free in groups 1 and 2, there were CIRF in 10 (29%) and 8 (20%) renal units in groups 1 and 2, respectively.

No major perioperative complications were seen. Some minor complications were recorded in 7 patients in each group (Table 2). Minor ureteral trauma occurred in 1 and 2 patients in groups 1 and 2, respectively. Intraoperative hemorrhage was seen in 1 case in each group. The procedures were not cancelled due to intraoperative complications and the operations were completed without any difficulty. Renal colic was detected in 3 and 2 patients in groups 1 and 2. Four patients with renal colic were treated with parenteral medications in the emergency setting in groups 1 and 2 (Clavien 2). On the other hand, internal stent was placed in one patient with renal colic in group 2 due to hydroureteronephrosis (Clavien 3b). Prolonged hematuria that lasted longer than a week was seen in one patient in each group and treated conservatively without any transfusion (Clavien 1). Urinary tract infection (UTI) was seen in one patient in each group. The patient with pyelonephritis was treated with parenteral medications after hospitalization in group 1 (Clavien 2). Another patient with UTI was treated with appropriate antibiotics in the outpatient setting (Clavien 2).

## 4. Discussion

Since urolithiasis is a recurrent disease, multiple interventions are often needed in these patients [3]. In the past, ORSS was commonly performed in the treatment of urinary stone disease. However, with the recent technological development, ESWL, PNL, and RIRS have replaced open surgery. Eventually PNL and RIRS are being utilized more commonly instead of open surgery in the treatment of many patients who had a previous ORSS because of secondary urolithiasis. While PNL is an effective treatment option performed for the management of large and complex calculi, it is more invasive, with a significant rate of major complications especially in secondary cases [4–6, 11]. According to the European Association of Urology (EAU) guidelines, although RIRS or ESWL is recommended as the first line treatment in the management of kidney stones <10 mm or 10–20 mm, RIRS is not recommended as a first line therapy for kidney stones >20 mm since SFRs are low and staged procedures may become necessary [2, 12]. However, RIRS can be successfully performed by experienced hands at high volume centers [13, 14]. Moreover recent studies have demonstrated that RIRS can be effective and safe as an alternative treatment option for relatively larger, multiple, bilateral, and ESWL refractory renal stones [15–18].

Although a lot of studies have addressed the specific issues regarding PNL in a kidney previously operated on, there is only one study specifically addressing RIRS after ORSS [4–8]. RIRS was performed in 53 patients who previously underwent ORSS by Osman and associates [8]. In their study, mean patient age, mean stone size, and mean stone number were 51 years (18–65), 14.3 mm (5–32), and 3.0 (2–8), respectively. This study mentioned that RIRS after previous ORSS had some challenges. First, accessing the kidney stone may be difficult because of fibrosis and distortion of the collecting system as a result of ORSS. Second, accessing the upper urinary system may be technically challenging because of bilharziasis, causing inflammatory distortion of the bladder and ureters, which is endemic in their country [8]. We did not detect any serious bladder or lower ureteral pathologies in our series. Access sheath was used in the 39 (95%) of 41 procedures. Flexible ureteroscopy without access sheath was done in the remaining 2 (5%) patients, who had mild ureteral stenosis. On the other hand, renal stones could have not been reached due to ureteropelvic stenosis in 2 patients who were successfully treated at 2 separate sessions: in the first session, endopyelotomy and internal stent placement were performed and in the second session flexible ureteroscopy and laser lithotripsy were performed. We encountered that there were infundibular strictures and distortions in the intrarenal collecting system and the stone was covered with fibrotic tissue in one patient. This patient was treated with two separate sessions: fibrotic tissue vaporization and partial laser fragmentation were performed in the first session; the remaining stone fragment was broken in the second session.

In the study by Osman et al., mean operative time, use of ureteral access sheath, and internal stent placement were 82 minutes (20–130), 41 (77%) patients, and 53 (100%) patients, respectively [8]. Their success rates were 79.2% and 92.4% after the first- and second-session RIRS. Our study was different only in internal stent placement rates compared with the above-mentioned study. All patients in their study received internal stent placement to prevent ureteral strictures since bilharziasis is endemic in their country. In our series, internal stent placement was done in 71% (29/41) cases in the study group. Moreover overall SFRs after first- and second-session procedures were 82% and 100% and 90% and 95% for study and control groups, respectively (*p* = 0.496). The success rates in both studies were similar to each other and to the previously published RIRS series [15–17]. Consequently, these two studies demonstrated that RIRS is an effective and safe option in the management of patients with renal stones who previously underwent ORSS.

According to the recent published literature the overall complication rates of RIRS are between 6% and 23% [8, 16–19]. Ureteral traumas, access problems, intraoperative hematuria, prolonged hematuria, urinary tract infections, and renal colic are the most encountered complications [16–18]. Osman and collages reported that overall complication rate after RIRS in patients who underwent ORSS previously was 20.7% [8]. They reported two intraoperative complications (ureteral perforation and bleeding); both were treated conservatively. Additionally, some postoperative complications including febrile UTI (Clavien 2), hematuria (Clavien 1), stone street (Clavien 3b), and anuria in the solitary kidney (Clavien 3b) were also reported in their study [8]. In our study, overall complication rates in both groups were 17% (*p* = 0.596) and were similar to the current literature. All complications but 1 (*n* = 7) in group 1 were conservatively treated at the outpatient setting. Pyelonephritis (Clavien 2) was observed in one patient who was treated with parenteral medication after being hospitalized. On the other hand, only one patient with renal colic (Clavien 3b) in group 2 was treated with internal stent placement. Our study showed that the complication rates and types of complication did not change after RIRS in patients with previous ORSS compared with that of the primary RIRS procedure.

Our study has some limitations. First, it is a retrospective, nonrandomized study from a single center. Second, there were small numbers of patients. In spite of these limitations, our study is the second study on RIRS in the treatment of patients who underwent ORSS previously.

## 5. Conclusion

RIRS in patients treated previously with ORSS can be safely and effectively performed with acceptable complication rates as in patients with no previous ORSS by the hands of experienced surgeons when essential endourological pieces of equipment are available.

## Figures and Tables

**Table 1:** Patient demographics and preoperative stone characteristics.

	Group 1 (study group)	Group 2 (control group)	*p* value
Gender (M/F)	24/08	26/12	0.603
Mean age (year)	40.5 ± 12.8 (20–72)	45.5 ± 12.6 (23–74)	0.110
Mean BMI (kg/m^2^)	28.1 ± 5.3 (19–42)	26.7 ± 4.1 (19–41)	0.217
Mean stone number (*n*)	2.7 ± 1.5 (1–7)	2.5 ± 1.3 (1–8)	0.544
Mean stone size (mm)	10.1 ± 5.6 (3–30)	10.3 ± 4.2 (3–21)	0.551
Mean stone burden (mm)	25.4 ± 14.7 (7–58)	23.5 ± 9.9 (6–60)	0.504
Total stone number (*n*)	106	101	
Localization (per procedure)			
Upper ureter	6 (5%)	10 (10%)	
Upper calyx	14 (13%)	11 (11%)	
Middle calyx	23 (22%)	18 (18%)	
Lower calyx	36 (34%)	42 (41%)	
Renal pelvis	27 (26%)	20 (20%)	
Lateralization (per patient)			
Right side	17 (53%)	13 (34%)	
Left side	13 (41%)	23 (61%)	
Bilateral	2 (6)	2 (5)	

**Table 2:** Intraoperative and postoperative comparisons between groups.

	Group 1 (study group)	Group 2 (control group)	*p* value
Mean operative time (min)	79.5 ± 37.8 (25–165)	76.1 ± 35.9 (30–150)	0.463
Use of ureteral access sheath	95% (39/41)	88% (37/42)	0.433
Use of basket catheter	56% (23/41)	52% (22/42)	0.827
Ureteral stent placement	71% (29/41)	67% (28/42)	0.814
Mean duration of ureteral stent (day)	30.3 ± 26.6 (2–120)	22.1 ± 9.2 (3–30)	0.127
Mean hospital stay (hour)	23.7 ± 8.0 (6–48)	25.6 ± 6.4 (18–48)	0.258
SFR (%)			
First procedure	82% (28/34)	90% (36/40)	0.497
Second procedure	100% (34/34)	95% (38/40)	0.496
Complication rates	17% (7/41)	17% (7/42)	0.596
(1) Intraoperative	5%	7%	
Ureteral trauma	1	2	
Hemorrhage	1	1	
(2) Postoperative	12%	10%	
Renal colic	3	2	
Prolonged hematuria	1	1	
Urinary tract infection	1	1	
